# Efficacy of high-intensity, low-volume interval training compared to continuous aerobic training on insulin resistance, skeletal muscle structure and function in adults with metabolic syndrome: study protocol for a randomized controlled clinical trial (Intraining-MET)

**DOI:** 10.1186/s13063-018-2541-7

**Published:** 2018-02-27

**Authors:** Jaime Gallo-Villegas, Juan Carlos Aristizabal, Mauricio Estrada, Luis H. Valbuena, Raul Narvaez-Sanchez, Jorge Osorio, Daniel C. Aguirre-Acevedo, Juan C. Calderón

**Affiliations:** 1 GRINMADE Group, University of Antioquia, Medellín, Colombia; 2 Epidemiology Group, University of Antioquia, Medellín, Colombia; 3 GRAEPIC Group, University of Antioquia, Medellín, Colombia; 4 SICOR Clinical and Research Center, Medellín, Colombia; 5 Physiology and Biochemistry Research Group-PHYSIS, Medellín, Colombia; 6 Pablo Tobón Uribe Hospital, Medellín, Colombia; 7 Indeportes Antioquia, Medellín, Colombia

**Keywords:** Metabolic syndrome, Exercise training, Interval training, Aerobic exercise, Skeletal muscle, Risk factors, Insulin resistance, Hyperinsulinemia, Glucose metabolism disorders, Myokines

## Abstract

**Background:**

Evidence of the efficacy of high-intensity, low-volume interval training (HIIT-low volume) in treating insulin resistance (IR) in patients with metabolic disorders is contradictory. In addition, it is unknown whether this effect is mediated through muscle endocrine function, which in turn depends on muscle mass and fiber type composition. Our aims were to assess the efficacy of HIIT-low volume compared to continuous aerobic exercise (CAE) in treating IR in adults with metabolic syndrome (MS) and to establish whether musclin, apelin, muscle mass and muscle composition are mediators of the effect.

**Methods:**

This is a controlled, randomized, clinical trial using the minimization method, with blinding of those who will evaluate the outcomes and two parallel groups for the purpose of showing superiority. Sixty patients with MS and IR with ages between 40 and 60 years will be included. A clinical evaluation will be carried out, along with laboratory tests to evaluate IR (homeostatic model assessment (HOMA)), muscle endocrine function (serum levels of musclin and apelin), thigh muscle mass (by dual energy x-ray absorptiometry (DXA) and thigh muscle composition (by carnosine measurement with proton magnetic resonance spectroscopy (^1^H–MRS)), before and after 12 weeks of a treadmill exercise program three times a week. Participants assigned to the intervention (*n* = 30) will receive HIIT-low volume in 22-min sessions that will include six intervals at a load of 90% of maximum oxygen consumption (VO_2_ max) for 1 min followed by 2 min at 50% of VO_2_ max. The control group (*n* = 30) will receive CAE at an intensity of 60% of VO_2_ max for 36 min. A theoretical model based on structural equations will be proposed to estimate the total, direct and indirect effects of training on IR and the proportion explained by the mediators.

**Discussion:**

Compared with CAE, HIIT-low volume can be effective and efficient at improving physical capacity and decreasing cardiovascular risk factors, such as IR, in patients with metabolic disorders. Studies that evaluate mediating variables of the effect of HIIT-low volume on IR, such as endocrine function and skeletal muscle structure, are necessary to understand the role of skeletal muscle in the pathophysiology of MS and their regulation by exercise.

**Trial registration:**

NCT03087721. High-intensity Interval, Low Volume Training in Metabolic Syndrome (Intraining-MET). Registered on 22 March 2017, retrospectively registered.

**Electronic supplementary material:**

The online version of this article (10.1186/s13063-018-2541-7) contains supplementary material, which is available to authorized users.

## Background

Metabolic syndrome (MS) is a frequent chronic condition related to physical inactivity, inadequate eating habits and obesity; MS increases the risk of type 2 diabetes mellitus and cardiovascular disease [1, 2]. The pathophysiology of MS is related to vascular and metabolic alterations, such as arterial hypertension, visceral fat accumulation, dyslipidemia, insulin resistance (IR), glucose intolerance and persistent increases in various pro-thrombotic, anti-fibrinolytic and inflammatory factors [3].

IR is a central component of MS associated with increased cardiovascular morbidity and mortality [2, 4]. IR increases the risk of diabetes (RR 6.9, 95% CI 3.7–13) and cardiovascular disease (RR 2.2, 95% CI 1.7–3.1) [4]. The increase in morbidity and mortality in patients with MS has required the application of preventive strategies and pharmacological and non-pharmacological treatment.

Aerobic exercise is a preventive and treatment strategy that reduces IR and modifies risk factors in patients with MS [5–7]. However, only half of the population complies with the current recommendation of 150 min per week of physical activity at a moderate intensity [8]. One of the main barriers to being more active perceived by people is the “lack of time” [9], which has motivated the search for the minimum amount of physical activity necessary to achieve health-related benefits and facilitate adherence [10].

High-intensity interval training (HIIT) has been used in the treatment of patients with cardio-metabolic diseases to increase adherence because it requires less volume. Compared to continuous aerobic exercise (CAE), this type of training is an effective alternative for improving maximal oxygen consumption (VO_2_ max), blood pressure, cardiac function, glucose and lipid metabolism and oxidative stress and inflammation markers [7, 11, 12]. However, the results regarding its effectiveness on IR in patients with MS and type 2 diabetes mellitus have been contradictory [6]: while one study reported greater effectiveness of HIIT than CAE in treating IR [5], others did not show differences between the two training strategies [13–15].

High-intensity, low-volume interval training (HIIT-low volume) is a type of HIIT that uses intensities close to the maximum in sessions of short duration. This training seems to generate greater local peripheral stimulation of skeletal muscle with the advantage of requiring less time and volume [11, 16, 17]. Currently, the evidence on its efficacy and safety is scarce; this evidence has been obtained from studies with quasi-experimental methodological designs that have included people without metabolic alterations, with the results obtained in response to a single exercise session or programs with duration less than 7 weeks [11, 16, 17].

Because HIIT-low volume can be demanding, not tolerable or unattractive to non-active people, training methods have been designed for high-risk populations with cardio-metabolic diseases [11, 16–18]. These methods incorporate some important elements, such as (i) decrease in the absolute intensity during the maximum load phase; (ii) increase in the stimulus duration from 30 s to 1 min; (iii) recovery periods between 1 and 2 min; and (iv) 20-min training sessions [11, 18]. With this protocol, an improvement in glucose metabolism was observed for 24 h after a two-week program in patients with diabetes mellitus [18]. Despite the short duration of the protocol, this type of training led to remodeling of the skeletal muscle and a more oxidative phenotype. It is possible that this effect was obtained by a greater muscular peripheral stimulus mediated through the peroxisome proliferator-activated receptor γ co-activator 1α (PGC-1α), a key driver of mitochondrial biogenesis and oxidative metabolism [11, 18, 19].

Among the molecular mechanisms that could explain a greater decrease in IR in response to HIIT-low volume compared to CAE are (i) a greater increase in the expression of PGC-1α; (ii) a greater increase in glucose uptake by skeletal muscle; (iii) a greater increase in the intramuscular expression of glucose transporter type 4 (GLUT-4); and (iv) greater insulin sensitivity (IS) related to greater depletion of intramuscular glycogen [6, 11, 18, 19].

Although the molecular mechanisms described could explain the greater effectiveness of HIIT-low volume compared to CAE on IR [11, 16, 18, 19], it is unknown whether the differences in this effect are mediated by the muscle endocrine function, which in turn depends on the quantity and composition of the skeletal muscle. Currently, there is some evidence that could support the potential mediating role of muscle endocrine function in the effect of HIIT-low volume on IR: (i) skeletal muscle is an endocrine organ involved in the pathophysiology of multiple chronic diseases [20, 21]; (ii) different myokines, such as irisin, myostatin, myonectin, interleukin-6 (IL-6), musclin and apelin, may be involved in IR, glucose uptake, oxidation of fats and production of hepatic glucose and lipolysis [20, 22]; (iii) lower muscle mass in the lower limbs, particularly in the thigh, is associated with MS, diabetes mellitus and increased cardiovascular morbidity and premature mortality [23, 24]; and (iv) the predominance of type II fibers observed mainly in obese people is related to IR [25].

Because exercise can decrease the expression of intramuscular musclin in murine models of IR [26], increase the serum concentrations of apelin in humans [27], and increase the muscle mass and modify the composition of the muscle fiber type towards a more oxidative phenotype [16], it is proposed that these variables may be potential mediators of the beneficial effect of HIIT-low volume on IR.

The present study aims to evaluate the efficacy of HIIT-low volume compared to CAE on IR in adults with MS and to establish whether musclin, apelin, mass and muscle fiber type of the thigh are mediators of the effect. For a hypothesis, we propose that HIIT-low volume is more effective in reducing IR and that the differences in the effect between the two training modalities are mediated by muscle endocrine function, which in turn depends on the amount and composition of fiber types.

## Methods

### Study design

**Fig. 1:**
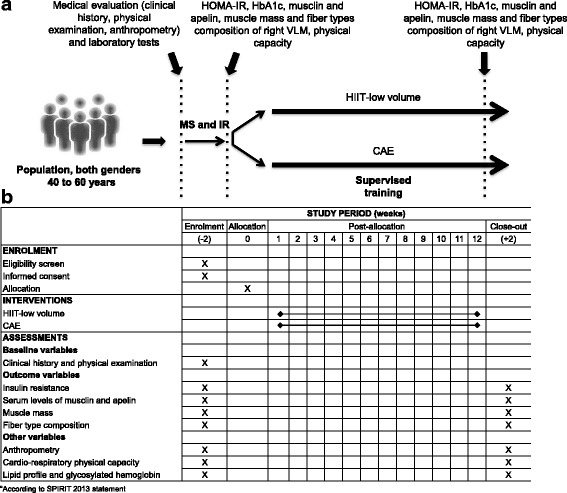
General design of the study, schedule of enrollment, intervention and assessment*. **a** After signing the informed consent, the patients will undergo medical and biochemical evaluation. Once inclusion/exclusion criteria are verified, recruited patients will be evaluated for muscle mass, fiber type composition of the *vastus lateralis* muscle (VLM), and cardio-respiratory physical capacity. Then, patients will be assigned to a high-intensity/low-volume interval training (HIIT-low volume) or continuous aerobic exercise training (CAE) intervention. A final reevaluation will be performed. **b** Evaluations during both enrollment and close out periods are expected to last 2 weeks. The intervention will last 12 weeks. HOMA-IR, Homeostatic Model Assessment-Insulin Resistance; HbA1c, glycated hemoglobin; IR, insulin resistance; MS, metabolic syndrome

A controlled, randomized clinical trial using the minimization method will be conducted, with blinding of those who will evaluate the outcomes and two parallel groups for the purpose of showing superiority. Sixty patients with MS and IR will be included and will undergo medical evaluation, laboratory tests, measurement of muscle mass and fiber type composition in the thigh, and cardio-respiratory physical capacity, both before and after 12 weeks of a treadmill exercise program. The participants assigned to the intervention will receive HIIT-low volume for 22 min, and the control group will receive CAE for 36 min three times per week. The study will be implemented and reported in line with Standard Protocol Items for Randomized Trials [28] (Fig. 1 and Additional file 1).

### Study site

This study will be carried out in the cardiac rehabilitation and physiotherapy section of the facilities of the outpatient clinic of the IPS-Universitaria, in Medellín (Colombia).

### Recruitment of participants

Volunteers will be recruited from the academic community of the University of Antioquia, associated institutions and in nearby communities, through messages sent by email or Facebook, posters located in various faculties, and through direct invitation to those who attend health promotion and cardiovascular prevention activities at the University. The expected time for the recruitment of participants will be 18 months. If the recruitment is slower than expected we will implement educational and communication strategies within the target populations, in order to increase the number of participants. Also, we can extend the recruitment period for another 6 months if needed. The recruitment will end once the number of required volunteers is obtained. There will be no incentives paid to participate in this study.

### Eligibility criteria

People aged between 40 and 60 years, with three criteria for MS [1], with IR according to the homeostatic model assessment (HOMA-IR) greater than 2.25 [29, 30] and with a sedentary lifestyle [31] will be included. People with a vegetarian diet, supplemental consumption of vitamin D3, injuries or musculoskeletal diseases that prevent exercise; people in a situation of physical, sensory and cognitive disability; people with a history of cardiopulmonary disease or acute or chronic inflammatory conditions, cancer, acquired immune deficiency syndrome or diabetes mellitus; and pregnant women will be excluded.

### Interventions

The training program will be controlled by physical activity specialists and will include three sessions per week of treadmill walking/running, with a progressive increase in training loads of 5% every 2 weeks and a total duration of 12 weeks.

#### High-intensity and low-volume interval training

**Fig. 2:**
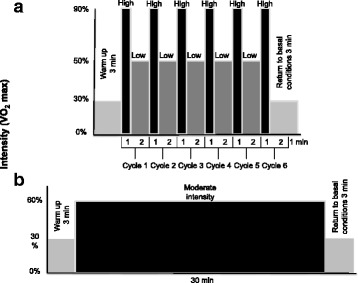
Training protocols for intervention. **a** High-intensity/low-volume interval training protocol scheme. **b** Continuous aerobic exercise protocol scheme. VO_2_ max, maximum oxygen consumption

The HIIT-low volume session will include a warm-up period of 3 min at an intensity of 30% of VO_2_ max (expressed as a measure of the external load in the speed and inclination of the treadmill), followed by six intervals that include 1 min of high intensity with a workload of 90% of VO_2_ max and 2 min with a workload of 50% of VO_2_ max. The session will end with a cooling off period of 3 min at an intensity of 30% of VO_2_ max for a total duration of 22 min (Fig. 2a).

#### Continuous aerobic exercise

The CAE session will include a warm-up period of 3 min at 30% of VO_2_ max, followed by 30 min at 60% of VO_2_ max; it will end with a cooling off period of 3 min at 30% of VO_2_ max for a total duration of 36 min (Fig. 2b).

### Adherence to the protocol

Adherence will be established from the quantification of a percentage, taking into account the total volume of physical activity performed in relation to the total volume of physical activity scheduled.

### Concomitant interventions

All people will continue with their pharmacological treatment plan and nutritional recommendations provided by the treating physician.

### Measurement of covariates and outcomes

For the collection of information, electronic formats will be designed to record the data related to each training session, the measurement of covariates and the outcomes.

#### Clinical history and physical examination

A complete clinical history will include socio-demographic information, habits and personal and family histories. Blood pressure and heart rate will be measured in the sitting and standing positions according to current recommendations [32]. For the quantification of physical activity, the Global Physical Activity Questionnaire (GPAQ) [33] validated in Colombia [34] and applied by our research group [23], will be used. The caloric intake will be estimated from the frequency of food consumption at the beginning and end of the study.

#### Anthropometry

Height will be measured with a Seca® 2013 stadiometer (Seca, Germany), and body weight will be measured with an Omron® HBF-510LA scale (Omron Healthcare, Inc., USA) with accuracy of 0.1 kg. Waist circumference will be measured with a fiberglass anthropometric tape at the intermediate point between the lower edge of the last rib and the iliac crest, in the horizontal plane. The perimeter of the thigh will be measured 1 cm below the gluteal fold, perpendicular to the longitudinal axis of the thigh. Body mass index (BMI) will be calculated with the formula weight (kg)/height (m)^2^.

#### Cardio-respiratory physical capacity

VO_2_ max will be measured directly with an open circuit spirometer Oxycon Delta by Jaeger® (VIASYS Healthcare GmbH, Germany) and a treadmill. An exercise physiologist will monitor the test, and the electrocardiogram and heart rate will be recorded continuously. Participants will initially warm up for 3 min, walking at a speed of 3.5 miles per hour, with an inclination angle of 1%. Then, the speed of the treadmill will increase 0.5 miles per hour every minute until exhaustion. It will be considered a maximum test when (i) a plateau in VO_2_ despite an increase in work or a respiratory quotient equal to or greater than 1.10 are observed; and (ii) the participants reach the expected maximum heart rate.

#### Lipid profile and glycosylated hemoglobin

A venous blood sample will be obtained after a fast of 8–12 h to measure the total cholesterol, high-density lipoprotein cholesterol (HDL), plasma triglycerides and glycosylated hemoglobin, using standardized procedures.

#### Insulin resistance

HOMA-IR will be used to estimate IR, insulin sensitivity (IS) and the percentage of functioning pancreatic β cells (% β) [35]. HOMA-IR will be calculated before and between 44 and 48 h after the last session of the exercise training program, from the blood glucose and insulinemia values obtained from a venous blood sample taken after fasting for 8–12 h [35]. The measurement of insulinemia will be performed with the sandwich immunoassay technique with direct chemiluminescence technology with an ADVIA Centaur® CP Immunoassay System (Siemens, Germany).

#### Quantification of serum levels of musclin and apelin

Concentrations of musclin and apelin will be measured in serum using a sandwich-type enzyme linked immunosorbent assay kit (Human Musclin ELISA kit, LifeSpan BioSciences Inc., Catalog number LS-F7799, USA; Human Total Apelin ELISA kit, MyBioSource, Catalog No. MBS-725907, USA). Samples will be taken before and between 44 and 48 h after the last exercise session. Initially, the samples will be stored at − 80 °C, and then for the assay, they will be thawed, and 100 μL will be applied in duplicate to the wells of the ELISA kit. The samples will be incubated at 37 °C; subsequently, the secondary antibody conjugated to biotin will be applied; finally, the samples will be incubated with avidin coupled to an enzyme with peroxidase action. The optical density of each well will be determined in an ELISA reader (Thermo Scientific Varioskan Lux, USA) at 450 nm. The final values will be reported in picograms/milliliter of musclin and apelin.

#### Measurement of muscle mass

This step will be performed at the beginning and at the end of the exercise training program, within the first 96 h after the last session, by employing the dual energy x-ray absorptiometry (DXA) technique using a Discovery Wi DXA system® (Hologic, USA) and the Hologic APEX v4.5.3 program (Hologic, USA). The measurement of thigh muscle mass will be made on the right side according to the technique described by Visser and defined by an area located between two horizontal lines located at the lowest point of the ischial tuberosity and at the knee joint as the upper and lower limits, respectively [36].

#### Evaluation of the fiber type composition of the right *vastus lateralis*

The estimation of the proportion of the area occupied by type II muscle fibers will be made at the beginning and the end of the exercise training program in the first week after the last session, based on the quantification of carnosine in the right *vastus lateralis* muscle (VLM) using the proton magnetic resonance spectroscopy technique (^1^H–MRS) [37] and a flexible coil according to the technique standardized by our group to evaluate body segments of large muscle mass [38], in turn modified from Baguet’s technique [37]. A three-Tesla Magnetom Skyra magnet, with a Flex Large 4A3T coil interface and the SyngoMR D13 program (Siemens, Germany), will be used. Each person will be placed in the supine position on the spectroscopy table, with the lower limbs extended and supported on a 5-cm cushion in the popliteal region. The largest mass of the VLM will be sought in the area between the middle of the externally facing right thigh and the middle third and distal junction of the same thigh; these anatomical points will be indicated with vitamin E capsules. A voxel of 10 × 15 × 35 mm will be positioned in the most voluminous region of the muscle, in the area delimited by the capsules, using a T1-weighted gradient-echo sequence in the three planes, with 5 mm sections and repetition time (RT)/echo time (ET) = 250/2.52 ms. The acquisition of the spectra will be performed using a point-resolved single voxel spectroscopy (PRESS) sequence with and without water saturation to evaluate the carnosine and water signals, respectively. For the processing of the signals and the absolute quantification of carnosine, the jMRUI 5.1 program (Autonomous University of Barcelona, Spain) will be used, with phase correction, Gaussian apodization, adjustment of the water reference to 4.7 ppm and subtraction of water, lipids and other metabolites within the signal.

### Sample size

A total required sample size of 30 people in each group was calculated assuming an IS increase of 10% [39], 15% standard deviation [5], a 95% confidence level, 80% power, a 1:1 ratio, and a dropout rate of 5%. In addition, a correlation coefficient of 0.44 between the baseline and final IS values was taken into account [40].

### Random assignment, implementation and masking

The Coordinating Center of the SICOR Clinical Trial, external to the group of researchers, will perform the randomization at a ratio of 1:1 (HIIT-low volume, *n* = 30; CAE, *n* = 30), with the minimization method [41] with Minimpy® software, version 0.3 [42], taking into account the values of the following variables: age (< 50 and ≥ 50 years), gender (male and female) and BMI (< 30 and ≥ 30 kg/m^2^). A base probability of 0.7 will be used under the “bias coin” method and variance as a measure of the imbalance distance between the groups, for the inclusion of the next participant [43].

The communication between the researchers who will perform the intervention and the center responsible for the assignment to the treatment groups will be conducted by telephone to guarantee independence. The measurement of outcomes will be carried out by evaluators who will not know the assignment of patients to the treatment groups. Two researchers will feed a database based on information recorded in formats at each training session, with the measurement of covariates and outcomes. The files of the participants will be stored in numerical order in a safe place and will be accessible through a password. A code will be used that will prevent identification of the patient and that, in turn, will not be visible to the person performing the statistical analysis.

### Statistical analysis

Initially, an exploratory analysis of the data will be carried out, and the Shapiro-Wilk test will be used to evaluate if the quantitative variables come from a population with a normal distribution. The mean and the standard deviation, the median and the interquartile range and the percentage will be used for the description of the socio-demographic, clinical, anthropometric and physiological variables of the included persons.

Both intention-to-treat and by-protocol analyses will be carried out; the latter will consider those patients who had adherence higher than 80%. The multiple imputation method (10 imputations) will be used to manage missing data based on age, gender, BMI and baseline VO_2_ max [44].

Analysis of covariance (ANCOVA) will be used [45] to estimate the effect of HIIT-low volume compared to CAE on IR, IS, percentage of pancreatic β-cell functioning, plasma levels of musclin and apelin and mass and composition of thigh muscle by fiber type at the end of the intervention. The assumptions of homogeneity of variance will be assessed with the Levene F test and the linearity and homogeneity of the slopes. If the assumptions are not met, a logarithmic transformation will be performed, or the Box-Cox procedure will be used. The variables used in the minimization procedure (age, gender and BMI) will be included in the analysis according to the current recommendations [41]. A re-randomization test will be performed using permutations for inference purposes [41].

**Fig. 3:**
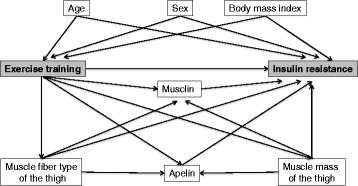
Conceptual model for the study of the effect of high-intensity/low-volume interval training or continuous exercise training on insulin resistance in patients with metabolic syndrome. The model considers mediator variables such as musclin and apelin, muscle mass and fiber type composition in the thigh; and confounding variables such as age, gender and body mass index

To establish if the musclin, apelin, muscle mass and fiber type composition of the thigh mediate the effect of training on IR, a theoretical model was proposed to estimate the total, direct and indirect effects and the percentage explained by the mediator (Fig. 3). A model of structural equations will be used with the “path analysis” technique and basic principles proposed in the context of clinical trials [46–48]. A statistical significance level œ = 0.05 will be established, and STATA, version 14.0, and IBM SPSS Statistics, version 21.0, will be used.

### Data monitoring

A data monitoring committee will not be formed because the duration of the intervention is short, the risk is minimal, and no interim analysis is required.

### Adverse events

The training modalities that are going to be used are safe; the reported frequencies of adverse events are very low and are mainly related to osteo-muscular injuries [6, 7]. The possible adverse osteo-muscular, metabolic, cardiovascular and other types of adverse events that occur during physical training will be monitored. The physical activity specialist in charge of the program will investigate and record the possible clinical manifestations related to the exercise.

### Audit

An independent Audit Committee will be made up of two professionals: a specialist in sports medicine and an epidemiologist. This committee will verify every 6 months the processes related to recruitment, informed consent, eligibility, assignment to the treatment groups and adherence to the intervention.

## Discussion

In recent years, different studies have shown the superiority of HIIT compared to CAE in the improvement of physiological and clinical variables in patients with different cardio-metabolic diseases [12]. In patients with MS, a greater increase in VO_2_ max (35% vs. 16%, respectively), flow-mediated dilation of the brachial artery, mitochondrial function and insulin receptor phosphorylation in the VLM [5] was observed.

The intensity of the HIIT-low volume program proposed in our study will be calculated from the VO_2_ max and will be expressed as an external load measurement (speed and inclination of the rolling treadmill), which could facilitate compliance with the prescription that is provided without the need to continuously measure oxygen consumption. The high intensity will be sub-maximal for 1 min at 90% of VO_2_ max, similar to that reported in one study [13], but higher than that used in others [5, 14, 15]; the volume of training and energy expenditure will be lower than those for CAE [11, 16–18]. Although the effect of HIIT-low volume on IR at a sub-maximum intensity of 90% of VO_2_ max has been evaluated in 6 weeks [13], we consider that from a physiological point of view, training of 12 weeks duration could generate a greater stimulus [11]. It is also necessary to take into account that intensity may be more important than volume in the effect of exercise on IR and metabolic adaptations of skeletal muscle in patients with MS and diabetes mellitus; however, the evidence is still limited [11, 16–18].

Skeletal muscle is an endocrine organ that secretes different factors called myokines [20, 22], which can be modified by exercise and mediate the effect on IR, depending on the intensity and duration of the stimulus [49]. Musclin is a myokine that increases IR in cellular and murine models [50]. Recent studies showed that mice lacking musclin have a lower physical capacity [51] and a decrease in musclin expression was also observed after exercise training in obese rats [26]. In humans, the response of musclin to physical training is not known; however, we believe that HIIT-low volume may be more effective than CAE in decreasing serum concentrations of musclin and could mediate the effect of exercise on IR.

Apelin is a myokine involved in the pathophysiology of obesity, IR, diabetes mellitus type 2 and cardiovascular function [52]. Currently, there is controversy about the response of apelin concentrations after exercise interventions [53, 54]. While a study that included patients with diabetes mellitus reported an increase in circulating apelin levels after a CAE program [53], another investigation showed no change after a similar exercise program [54]. In obese men, an HIIT program and a muscle strengthening training program showed an increase in circulating levels of apelin; however, no differences were observed between the two training modalities [27]. We believe that HIIT-low volume may be more effective than CAE in increasing serum concentrations of apelin.

It is recognized that lower thigh muscle mass is associated with metabolic diseases and premature mortality [23, 24] and that the predominance of type II fibers, which is mainly observed in obese people, is related to IR [25]. The findings were inconsistent when body composition and muscle mass were evaluated with DXA before and after a HIIT program [13, 55]. In postmenopausal women, no differences were observed between those assigned to a HIIT program compared to those assigned to a muscle-strengthening program [55]. In men at risk of IR, HIIT, unlike CAE, led to a reduction in body mass, percentage of fat, fat mass and lean mass [13].

The concentration of muscle carnosine is related to the area occupied by type II muscle fibers, the predominant type of fiber in people with metabolic diseases [25]. In turn, the concentration of carnosine in the VLM of the quadriceps is related to IR in sedentary adults [56] and with greater buffer capacity in athletes [57]. It has been reported that muscle carnosine concentrations do not change after HIIT in athletes [58]; however, the adaptive response to exercise is different depending on the level of physical activity and the presence of co-morbidities.

Compared to CAE, HIIT-low volume produces faster adaptations in muscle remodeling, a greater increase in mitochondrial capacity and the activity of peroxisome proliferator-activated receptor γ, leading to a more oxidative phenotype [11]. These adaptations can be regional depending on the muscle groups used during training [11, 16–18]. For this reason, we expect that the HIIT-low volume is more effective than CAE in increasing or maintaining thigh muscle mass and generating a predominant muscle composition of type I fibers. In addition, it is unknown whether both variables (amount and composition of the thigh muscle by fiber type) mediate an indirect effect of HIIT-low volume on IR or whether this effect occurs through the muscle endocrine function (musclin and apelin). To the best of our knowledge, there are still no studies evaluating this hypothesis.

The study of mediating variables such as muscle endocrine function and the amount and composition by fiber type could broaden our knowledge about the causal pathways and mechanisms amenable to intervention through health promotion and disease prevention strategies [59]. The conceptual model and the approach of the mediating variables proposed by our group is innovative; we hope that it will provide information for explaining the effect of HIIT-low volume on IR. The mediating variables studied may be involved in the pathophysiology of surrogate or hard clinical outcomes (IR, cardiovascular morbidity and mortality). Currently, to the best of our knowledge, there are no studies that have evaluated potential mediating variables related to endocrine function and skeletal muscle structure in the effect of HIIT-low volume on IR in patients with MS.

## Trial status

The call for participants in the Intrainning-MET study began on 1 March 2017. We had enrolled 25 volunteers by submission of the manuscript. Of those, 19 have finished the intervention and one withdrew for personal reasons. The remaining volunteers are actively participating. Recruitment is expected to finish by September 2018. Data analysis will be completed by January 2019.

## Additional file

Additional file 1: SPIRIT 2013 Checklist: recommended items to address in a clinical trial protocol and related documents. (DOC 130 kb)

## Abbreviations

% β: Percentage of function of pancreatic β cells
^1^H-MRS: Proton magnetic resonance spectroscopy
ANCOVA: Analysis of covariance
BMI: Body mass index
CAE: Continuous aerobic exercise
DXA: Dual energy x-ray absorptiometry
ELISA: Enzyme-linked immunosorbent assay
ET: Echo time
GLUT-4: Glucose transporter type 4
GPAQ: Global Questionnaire on Physical Activity
HDL: High-density lipoprotein
HIIT: High-intensity interval training
HIIT-low volume: High-intensity/low-volume interval training
HOMA-IR: Insulin resistance by homeostatic model assessment
IL-6: Interleukin 6
IR: Insulin resistance
IS: Insulin sensitivity
LDL: Low-density lipoprotein
MS: Metabolic syndrome
PGC-1a: Peroxisome proliferator-activated receptor γ co-activator 1a
PRESS: Point resolved single voxel spectroscopy
RT: Repetition time
VO_2_ max: Maximum oxygen consumption

## References

[1] Alberti KG, Eckel RH, Grundy SM (2009). Harmonizing the metabolic syndrome: a joint interim statement of the international diabetes federation task force on epidemiology and prevention; National Heart, Lung, and Blood Institute; American Heart Association; World Heart Federation; International Atherosclerosis Society; and International Association for the Study of obesity. Circulation..

[2] Ford ES (2005). Risks for all-cause mortality, cardiovascular disease, and diabetes associated with the metabolic syndrome: a summary of the evidence. Diabetes Care..

[3] Reaven G (2002). Metabolic syndrome: pathophysiology and implications for management of cardiovascular disease. Circulation..

[4] Meigs JB, Rutter MK, Sullivan LM (2007). Impact of insulin resistance on risk of type 2 diabetes and cardiovascular disease in people with metabolic syndrome. Diabetes Care..

[5] Tjonna AE, Lee SJ, Rognmo O (2008). Aerobic interval training versus continuous moderate exercise as a treatment for the metabolic syndrome: a pilot study. Circulation..

[6] Jelleyman C, Yates T, O'Donovan G (2015). The effects of high-intensity interval training on glucose regulation and insulin resistance: a meta-analysis. Obes Rev..

[7] Weston KS, Wisloff U, Coombes JS (2014). High-intensity interval training in patients with lifestyle-induced cardiometabolic disease: a systematic review and meta-analysis. Br J Sports Med..

[8] Centers for Disease Control and Prevention. State Indicator Report on Physical Activity, 2010. Atlanta: U.S. Department of Health and Human Services; 2010.

[9] Korkiakangas EE, Alahuhta MA, Laitinen JH (2009). Barriers to regular exercise among adults at high risk or diagnosed with type 2 diabetes: a systematic review. Health Promot Int..

[10] Eijsvogels TM, Molossi S, Lee DC (2016). Exercise at the extremes: the amount of exercise to reduce cardiovascular events. J Am Coll Cardiol..

[11] Gibala MJ, Little JP, Macdonald MJ (2012). Physiological adaptations to low-volume, high-intensity interval training in health and disease. J Physiol..

[12] Kessler HS, Sisson SB, Short KR (2012). The potential for high-intensity interval training to reduce cardiometabolic disease risk. Sports Med..

[13] Earnest CP, Lupo M, Thibodaux J (2013). Interval training in men at risk for insulin resistance. Int J Sports Med..

[14] Mitranun W, Deerochanawong C, Tanaka H (2014). Continuous vs interval training on glycemic control and macro- and microvascular reactivity in type 2 diabetic patients. Scand J Med Sci Sports..

[15] Hollekim-Strand SM, Bjorgaas MR, Albrektsen G (2014). High-intensity interval exercise effectively improves cardiac function in patients with type 2 diabetes mellitus and diastolic dysfunction: a randomized controlled trial. J Am Coll Cardiol..

[16] Hood MS, Little JP, Tarnopolsky MA (2011). Low-volume interval training improves muscle oxidative capacity in sedentary adults. Med Sci Sports Exerc..

[17] Whyte LJ, Gill JM, Cathcart AJ (2010). Effect of 2 weeks of sprint interval training on health-related outcomes in sedentary overweight/obese men. Metabolism..

[18] Little JP, Gillen JB, Percival ME (2011). Low-volume high-intensity interval training reduces hyperglycemia and increases muscle mitochondrial capacity in patients with type 2 diabetes. J Appl Physiol (1985)..

[19] Little JP, Safdar A, Cermak N (2010). Acute endurance exercise increases the nuclear abundance of PGC-1alpha in trained human skeletal muscle. Am J Physiol Regul Integr Comp Physiol..

[20] Pedersen BK, Febbraio MA (2012). Muscles, exercise and obesity: skeletal muscle as a secretory organ. Nat Rev Endocrinol..

[21] Doherty TJ (2003). Invited review: aging and sarcopenia. J Appl Physiol (1985)..

[22] Pedersen BK (2011). Muscles and their myokines. J Exp Biol..

[23] Londono FJ, Calderon JC, Gallo J (2012). Association between thigh muscle development and the metabolic syndrome in adults. Ann Nutr Metab..

[24] Heitmann BL, Frederiksen P (2009). Thigh circumference and risk of heart disease and premature death: prospective cohort study. BMJ..

[25] Tanner CJ, Barakat HA, Dohm GL (2002). Muscle fiber type is associated with obesity and weight loss. Am J Physiol Endocrinol Metab..

[26] Yu J, Zheng J, Liu XF (2016). Exercise improved lipid metabolism and insulin sensitivity in rats fed a high-fat diet by regulating glucose transporter 4 (GLUT4) and musclin expression. Braz J Med Biol Res..

[27] Nikseresht M, Hafezi Ahmadi MR, Hedayati M (2016). Detraining-induced alterations in adipokines and cardiometabolic risk factors after nonlinear periodized resistance and aerobic interval training in obese men. Appl Physiol Nutr Metab..

[28] Chan AW, Tetzlaff JM, Gotzsche PC (2013). SPIRIT 2013 explanation and elaboration: guidance for protocols of clinical trials. BMJ..

[29] Gallo J, Ochoa J, Balparda J (2013). Puntos de corte del perímetro de la cintura para identificar sujetos con resistencia a la insulina en una población colombiana. Acta Med Colomb..

[30] Alberti KG, Zimmet PZ (1998). Definition, diagnosis and classification of diabetes mellitus and its complications. Part 1: diagnosis and classification of diabetes mellitus provisional report of a WHO consultation. Diabet Med..

[31] Ricciardi R (2005). Sedentarism: a concept analysis. Nurs Forum..

[32] Mancia G, De Backer G, Dominiczak A (2007). 2007 Guidelines for the management of arterial hypertension: the task force for the management of arterial hypertension of the European Society of Hypertension (ESH) and of the European Society of Cardiology (ESC). Eur Heart J..

[33] Bull FC, Maslin TS, Armstrong T (2009). Global physical activity questionnaire (GPAQ): nine country reliability and validity study. J Phys Act Health..

[34] Gomez LF, Duperly J, Lucumi DI (2005). Physical activity levels in adults living in Bogota (Colombia): prevalence and associated factors. Gac Sanit..

[35] Wallace TM, Levy JC, Matthews DR (2004). Use and abuse of HOMA modeling. Diabetes Care..

[36] Visser M, Fuerst T, Lang T (1999). Validity of fan-beam dual-energy X-ray absorptiometry for measuring fat-free mass and leg muscle mass. Health, aging, and body composition study–dual-energy X-ray absorptiometry and body composition working group. J Appl Physiol (1985)..

[37] Baguet A, Everaert I, Hespel P (2011). A new method for non-invasive estimation of human muscle fiber type composition. PLoS One..

[38] Estrada M, Vega G, Reyngoudt H (2016). Intramuscular absolute carnosine quantification in young athletes by 1H-MRS using a flexible coil. Skelet Radiol..

[39] Matinhomaee H, Banaei J, Ali M (2014). Effects of 12-week high-intensity interval training on plasma visfatin concentration and insulin resistance in overweight men. J Exerc Sci Fit..

[40] Gallo J, Pérez-Idárraga A, Valencia K (2016). Effect of dancing and nutrition education on hemodynamic and autonomic status in adults with metabolic syndrome: a randomized controlled clinical trial. Rev Colomb Cardiol..

[41] Scott NW, McPherson GC, Ramsay CR (2002). The method of minimization for allocation to clinical trials. A review. Control Clin Trials..

[42] Saghaei M, Saghaei S (2011). Implementation of an open-source customizable minimization program for allocation of patients to parallel groups in clinical trials. J Biomed Sci Eng..

[43] Pocock SJ, Simon R (1975). Sequential treatment assignment with balancing for prognostic factors in the controlled clinical trial. Biometrics..

[44] Newgard CD, Haukoos JS (2007). Advanced statistics: missing data in clinical research–part 2: multiple imputation. Acad Emerg Med..

[45] Egbewale BE, Lewis M, Sim J (2014). Bias, precision and statistical power of analysis of covariance in the analysis of randomized trials with baseline imbalance: a simulation study. BMC Med Res Methodol..

[46] Baron RM, Kenny DA (1986). The moderator-mediator variable distinction in social psychological research: conceptual, strategic, and statistical considerations. J Pers Soc Psychol..

[47] Hafeman DM, Schwartz S (2009). Opening the black box: a motivation for the assessment of mediation. Int J Epidemiol..

[48] Kraemer HC, Wilson GT, Fairburn CG (2002). Mediators and moderators of treatment effects in randomized clinical trials. Arch Gen Psychiatry..

[49] Pedersen BK, Febbraio MA (2008). Muscle as an endocrine organ: focus on muscle-derived interleukin-6. Physiol Rev..

[50] Nishizawa H, Matsuda M, Yamada Y (2004). Musclin, a novel skeletal muscle-derived secretory factor. J Biol Chem..

[51] Subbotina E, Sierra A, Zhu Z (2015). Musclin is an activity-stimulated myokine that enhances physical endurance. Proc Natl Acad Sci USA..

[52] Castan-Laurell I, Dray C, Knauf C (2012). Apelin, a promising target for type 2 diabetes treatment?. Trends Endocrinol Metab..

[53] Kadoglou NP, Vrabas IS, Kapelouzou A (2012). The impact of aerobic exercise training on novel adipokines, apelin and ghrelin, in patients with type 2 diabetes. Med Sci Monit..

[54] Besse-Patin A, Montastier E, Vinel C (2014). Effect of endurance training on skeletal muscle myokine expression in obese men: identification of apelin as a novel myokine. Int J Obes..

[55] Grossman J, Payne E (2016). A randomized comparison study regarding the impact of short-duration, high-intensity exercise and traditional exercise on anthropometric and body composition measurement changes in post-menopausal women–a pilot study. Post Reprod Health..

[56] de Courten B, Kurdiova T, de Courten MP (2015). Muscle carnosine is associated with cardiometabolic risk factors in humans. PLoS One..

[57] Parkhouse WS, McKenzie DC (1984). Possible contribution of skeletal muscle buffers to enhanced anaerobic performance: a brief review. Med Sci Sports Exerc..

[58] Edge J, Eynon N, McKenna MJ (2013). Altering the rest interval during high-intensity interval training does not affect muscle or performance adaptations. Exp Physiol..

[59] Richiardi L, Bellocco R, Zugna D (2013). Mediation analysis in epidemiology: methods, interpretation and bias. Int J Epidemiol..

